# Factors influencing decision to seek health care: a qualitative study among labour-migrants’ wives in northern Tajikistan

**DOI:** 10.1186/s12884-018-2166-6

**Published:** 2019-01-07

**Authors:** Jamila Nabieva, Aurélia Souares

**Affiliations:** Heidelberg Institute of Global Health, University of Heidelberg, Im Neuenheimer Feld 130/3, 69120 Heidelberg, Germany

**Keywords:** Tajikistan, Women’s health, Antenatal and obstetric care, Three delays model, Labour migration, Access to care

## Abstract

**Background:**

This qualitative study aimed to investigate the health seeking behaviour of rural women in northern Tajikistan, with specific focus on antenatal and obstetric complications as a result of delayed access to health services. Due to the unprecedented level of labour migration among men in the border region of Tajikistan, Isfara, the study specifically focused on migrants’ wives residing with their in-laws.

**Methods:**

Using an adapted “Three delays” model which suggests three major causes of delayed access to maternity services - ***decision to seek care****,* ***reaching a healthcare facility*** and ***receiving necessary care*** – we conducted 29 in-depth interviews with labour migrants’ wives, 16 semi-structured interviews with healthcare service providers and 2 focus-group discussions with 16 mothers-in-law in Isfara district.

**Results:**

Our study demonstrated that the most crucial and conditioned factor of access to maternity services for labour migrants’ wives is a decision to seek care. While ***reaching a healthcare facility*** (geographical accessibility, time and transportation costs) and ***receiving necessary care*** (availability of services, financial affordability and perceived quality of care) were rarely reported as obstacles towards timely access to maternity services, ***decision to seek care*** was found to be an intricate interplay of several factors: traditional gender and family roles (particularly in the absence of the husband), the age of the mother-in-law, cultural beliefs and perceptions about pregnancy and maternity, and widely spread myths about certain health conditions and services.

**Conclusions:**

Our study concludes that the traditional pattern of seeking health care among women in northern Tajikistan may often cause delays in accessing necessary maternity services and result in adverse health outcomes for women. We suggest that effective strategies to reduce maternal morbidity and mortality in rural Tajikistan should, along with strengthening healthcare structures, embark on community education and awareness raising with special focus on mothers-in-law and other traditional decision-makers in households.

## Background

Globally, women continue to face inequities in health care with regards to their sexual and reproductive health. About 830 women worldwide died every day from complications related to pregnancy and childbirth in 2015 [1] and over 90% of these deaths occurred in low-income countries. About 225 million women worldwide are estimated to have unmet family planning needs, 12.7 million of which are adolescent girls who are married or in union [2]*.* An estimated 100.000 deaths a year could have been prevented had timely access to effective contraception been available.

Access to care as the cornerstone idea of the Universal Health Coverage is often associated with determinants such as availability, accessibility, affordability and acceptability [3–5]*.* The gender determinant, namely being a woman, brings in additional social and cultural dimensions of access *to* and utilization *of* health care services. On the one hand, domestic and intimate partner violence, system discrimination and cultural harmful practices, such as female genital mutilation or female infanticide, pose gender-specific risks to women’s health and well-being. On the other hand, limited access to education, job market or adequate resources often prevents women from obtaining necessary healthcare services.

Tajikistan is a landlocked country in Central Asia with a population of 9 million, a 0.99/1 male/female ratio, 25% of female population in reproductive-age (15–49 years old), a total fertility rate of 3.8 and a 99% total literacy rate (2018). Within the first two decades after gaining independence from the Soviet Union in 1991, Tajikistan underwent significant demographic and health changes. The maternal mortality rate doubled in the first three years reaching an unprecedented level of 120 deaths per 100.000 live births in 1995, and later decreased to 44 by 2013 [6]*.* Girls’ net attendance rate to mandatory secondary school had decreased from 100% in 1990 to 70% in 2012, and back to 90% in 2017, reported adolescent marriage rate for girls (married before 18) reached 13%, with 85% of married adolescent girls justifying wife-beating [7]. The 2012 Tajikistan Demographic and Health Survey [8] showed that, in the context of access to necessary care during pregnancy, 45% of women stated that obtaining money from family members to cover healthcare costs was a major barrier; for 26–29% of women distance and unwillingness to travel alone was a serious obstacle, while 17% said that obtaining permission to see a healthcare provider was difficult.

The recent, 2017 Tajikistan Demographic and Health Survey [9] revealed that 92% of pregnant women sought professional care at least once during pregnancy, while 64% had 4 antenatal visits; 95% of women delivered with skilled birth attendance, 88% of which in a health facility.

## Labour migrants’ wives

A continuously declining economy and growing poverty have driven the majority of the country’s men to long-term labour migration to the Russian Federation, making Tajik households primarily dependent on remittances [10–14]. The 2009 Tajikistan Living Standards Measurement Survey showed that 9% of the country’s population was abroad because of labour migration, and 28% of households reported at least one family member had migrated [15]. The long-lasting migration of men has, in its turn, changed the lives and conditions of their wives left behind; women traditionally residing with their in-laws have limited or no control over family budget. Similarly, they often have limited autonomy to take decisions about their health and well-being [13].

Despite the persistent socio-economic and cultural developments aggravating women’s opportunities and living conditions, very limited research on women’s health and well-being has been conducted in Tajikistan.

With this qualitative study, we aimed to explore factors affecting women’s health seeking behaviour in rural Tajikistan; a special focus was made on access to antenatal and obstetric services. Our preliminary hypothesis was “*wives of labour migrants have limited opportunities to take decisions about access to health care which often leads to delays in obtaining necessary services”.* We deliberately selected labour migrants’ wives because their responses could serve a double purpose: (a) to describe common trends and barriers to access health care in disadvantaged groups of women; (b) to explore general health beliefs and practices among women in rural Tajikistan.

## Methods

### Study setting

This study was conducted in Isfara district of Sughd region, Tajikistan, which has an estimated 90% male labour migration rate. According to local authorities, these men are occupied in different seasonal jobs in the Russian Federation for at least 7 months every year; a big proportion of labour migrants stay abroad for two and more consecutive years. The long absence of men has gradually affected the living conditions and opportunities of their wives, who in most cases are unemployed or have no alternative sources of income. Traditional patterns of family relations and household management in rural Tajikistan oblige married women to reside with their in-laws, especially in the absence of their husbands [16–18]*.* The 2009 study on the abandoned wives of Tajik labour migrants [13] reported that women residing with their extended families often become economically and socially vulnerable. The latter creates an additional limitation for women to access necessary care: with 63$ per capita public spending on health (2015) and no health insurance system, informal out-of-pocket payments by service users are estimated to constitute over 70% of the total healthcare expenditures in Tajikistan [19]*.*

### Study design

In order to understand *how* decisions to seek health care are made and *why*, we opted for a qualitative research method with a descriptive and interpretive approach. To include cultural factors affecting women’s health choices and practices, we have introduced behavioural components in the data collection tools.

To ensure the validity and credibility of the collected data, we based our methodology on the triangulation of data sources and data collection tools: (a) in-depth interviews (IDI) with women provided primary information on women’s own experience accessing health care and the barriers to access; (b) semi-structured interviews (SSI) with healthcare professionals added more specific information on assumed barriers and actual practices; (c) focus-group discussions (FGD) with household leads highlighted common health beliefs and practices in the community.

### Conceptual framework

We have adapted the “Three delays” model by Thaddeus and Maine [20] originally designed to identify factors contributing to maternal death in the case of an obstetric complication from the moment of its onset to its eventual outcome. The authors suggest that a delay in access to necessary care may occur at any of the three stages: (1) ***decision to seek care;*** (2) ***reaching a healthcare facility*** and (3) ***receiving necessary care****.* They presume that the ***decision to seek health care*** can depend on factors such as costs, health beliefs, women’s power and autonomy to take decisions and can cause the first delay in accessing necessary obstetric services. Assuming a timely taken decision, physically ***reaching a healthcare facility*** can act as another delay, mainly due to the distance to health providers, transportation costs or road conditions. Finally, after reaching a healthcare provider, ***receiving necessary care*** in a timely and adequate manner can be delayed as the result of long waiting times and poor organization at a healthcare facility, a lack of necessary competences and supplies, or corruption, resulting in an adverse outcome of the complication.

The initial “Three delays” model has in the last two decades been widely expanded and adapted in different areas of health research and practice [21–24]*.* For the purposes of our study, we have adjusted the original model to investigate factors affecting access to health care services in general and built our questions on participants’ last episode of sickness. The adapted model was also used to explore the actual experience of women in accessing and obtaining maternity services in cases where a pregnancy occurred in the last three years.

### Sample selection

We purposely selected three study groups: (1) women from Isfara district whose husbands were in a labour migration for a period of over 6 months; (2) extended family members with household lead roles (mothers-in-law); (3) healthcare professionals in Isfara district providing antenatal, obstetric and postnatal services in primary, secondary and tertiary level facilities. Altogether, we conducted 29 in-depth interviews with migrants’ wives, 16 semi-structured interviews with healthcare providers and 2 focus group discussions with 16 mothers-in-law. We ensured that participants for in-depth interviews and focus group discussions were not related and did not belong to the same family or household.

We selected three villages (Navgilem, Surh, Kulkand) with different (a) socio-economic status; (b) levels of cultural and religious conservatism and (c) distance to the administrative centre where secondary and tertiary healthcare facilities are located. Unlike specialized secondary care, primary healthcare facilities exist in every village of Isfara district regardless of their size and distance from the administrative centre. Antenatal services as part of the primary health care are jointly provided by family doctors (for normal pregnancies) and gynaecologists (for pregnancies with complications). Obstetric care is available in the administrative centre, which for different villages of Isfara district is located up to 45 km away. For the three selected villages this distance ranges from 4 km (Navgilem) to 9 km (Kulkand) and to 15 km (Surh). Based on a Soviet example of a socialized, state-funded system, health care in Tajikistan is formally free for the population; in reality, due to extremely scarce state allocations for the healthcare system and the poor remuneration of medical professionals, most healthcare services, especially laboratory tests and medications, are covered by patients’ out-of-pocket, unofficial payments [8]*.*

### Data collection tools

Two interview guides and one FGD guide were applied to collect data. The in-depth interview guide for migrants’ wives and the FGD guide for mothers-in-law covered similar sets of topics to investigate a process of taking a decision to seek and access healthcare services for different household members. A semi-structured interview guide for healthcare professionals used antenatal and obstetric complications as a key theme to explore factors affecting women’s health-seeking practices in Isfara district.

The data collection tools were designed by both authors in the English language; approved tools together with consent forms were translated into Tajik and Russian languages. The first author conducted interviews and focus group discussions in either colloquial Tajik or Russian, depending on participants’ preferences. Written consent was obtained from interview participants (migrants’ wives and healthcare professionals); focus group discussion participants were asked for verbal consent that was tape-recorded before the start of the group discussions. All interviews and focus group discussions were tape-recorded with the permission of participants.

### Data management and analysis

The first author transcribed the data in the language it was originally collected in – Tajik or Russian. Given clear study objectives, the main themes and sub-themes in the data collection tools were used as preliminary codes complemented by new themes that emerged during analysis. The transcripts were analysed using a qualitative data analysis software: NVivo Qualitative Data Analysis Software; QSR International Pty Ltd. Version 10, 2012.

The collected data were first analysed against the study groups they were collected in. Further, codes that evolved across two or three groups were categorized as main themes. Simultaneously, new themes were put into separate categories based on their significance and relevance. While reducing categories, we were guided by the study objectives. Finally, we have compared the major findings in all three groups to identify links in similar categories across the groups. We based our interpretation of data on the preliminary hypothesis and the authors’ knowledge of the study context.

## Results

### Study group 1: Labour migrants’ wives

In-depth interviews were conducted with 29 women aged between 19 and 58 in Navgilem, Surh and Kulkand villages of the Isfara district. Table 1 presents summarized characteristics of study group 1.

**Table 1:** Characteristics of study group 1: Labour migrants’ wives

		N	%	Min	median	max
Age		29	100.0	19	32	58
Education	secondary school	29	100.0			
Occupation/job	housewife	26	89.7			
	retired housewife	1	3.4			
	midwife	1	3.4			
	maternity leave	1	3.4			
Years in marriage		29	100.0	0.5	13	36
Number of children		29	100.0	0	3	5

#### Health beliefs and behaviour

To understand participants’ assumptions on *locus of control* in the context of health and illness, women were asked what an individual’s health depends on. All respondents stated at least two or a combination of several factors among which economic opportunities and stress were mentioned most often. However, all respondents stated individual responsibility for one’s health as primary.

*“Being healthy or sick depends on the individual. But some diseases are because of stress: there are some men who can’t find a good job and feed their families … then women suffer emotionally [ … ] These thoughts about life make women sick [ … ]” –* Housewife, 32 years old*.*

All participants named medical professionals as their *preferred health providers* and reported never using traditional healers or anything beyond conventional medicine. But later in the interviews most of the women mentioned that they occasionally used home-made herbal treatments or religious healing rituals performed by the elderly in the household for conditions such as sore throat, diarrhoea or fever.

*Self-prescription* of over-the counter medicines is common, but limited to some mild symptoms such as fever, headache or tooth pain, and to medicines such as Paracetamol and Aspirin.

With high rates of out-of-pocket payments for health services in Tajikistan, families generally do not have secure *healthcare funds*; the necessary amount is mobilized as the need for healthcare services occurs. Pregnancy-related costs, e.g. potential antenatal complications or hospitalization, are usually not taken into consideration.

*“My husband works and sends money to his mother and me. So, when I need to see a doctor, I use that money. If that’s not enough, he* [husband] *tells me to borrow from my father-in-law, and he* [*husband*] *will earn and give it back to his father”* *–* Housewife, 33 years old.

#### Decision to seek care

We found that *permission* to seek health care services is a crucial factor for taking a decision, but most participants could not explain if this permission was about leaving the house, seeing a doctor or using funds for healthcare services. Women justified this practice as *respect* and said that permission was sought from at least two family members, with the mother-in-law and the husband mentioned primarily.

*“First I ask* *[permission]* *from my mother-in-law and father-in-law, since my husband said, they are the bosses. If they don’t object, then I call my husband. He says, if I have permission from his parents, he doesn’t mind me going to a doctor”* – A 32-year old housewife.

A *hierarchy* of decision-makers in the family was found to be common: during the periods when the husbands are in labour migration, women first contact the mothers-in-law and then call the husbands abroad. Women who no longer reside with their in-laws still seek the mother-in-law’s permission to see a doctor regardless of the fact whether their husbands are in the country or abroad. Women expressed a generalized preference for a *family council* as the best way to take decisions about an individual’s healthcare needs and treatment. Involvement of household leads in the decision-making process is believed to ensure financial and logistical support.

*“I think it’s always better to discuss it with the family members. But sometimes I feel shy to bring up some issues in front of men. Then I talk to my mother-in-law”–* A 21-year old housewife, married for 3 years.

#### Reaching a healthcare facility

None of the respondents reported facing difficulties with reaching a healthcare facility, be it transportation means, related costs, road conditions or distance. Primary healthcare facilities exist in all villages and are easily reachable within walking distance.

*“There is no problem in seeing a doctor, except that I sometimes get so busy that [I] completely forget about my health [ … ]” –* Housewife, 39 years old.

Secondary and specialized care is provided in the administrative centre, Isfara town, which in the case of the most remote village is 40–50 min away by car. The most common public transport is mini-buses, which run regularly and at a very affordable fare; many women also mentioned an automobile owned by their household members.

*“When my children don’t feel well, I always take them to our village doctor, he prescribes treatment [ … ] But sometimes his treatment doesn’t help, then I take my children to a doctor in town [ … ]” –* Housewife, 32 years old.

#### Receiving necessary care

All women reported that they were satisfied with the primary health care they received in the village level health centres; no difficulties in or obstacles to receiving care were mentioned. When referred to secondary, tertiary or specialized services (laboratory tests, advanced diagnostics) in Isfara town, women reported issues such as (a) time: travelling to town and queuing may impact women’s decision to see a doctor; (b) costs: services in specialized facilities in town cost more compared with village healthcare facilities, hence some women chose not to follow the referral if the case was not perceived as severe. Prescribed follow-up visits after the treatment by any facility level are often seen as unnecessary.

*“My doctor told me to see him after I finish the prescribed treatment. But I didn’t go [ … ] you know how it is – you are worried when you are sick. Once you feel better, it is no longer important [ … ]”* – A 33-year old housewife, 12 years in marriage.

#### Last pregnancy

Respondents who were pregnant in the previous three years reported being covered by antenatal care provided at village level Reproductive Health Centres (RHC). They had at least four antenatal visits, were aware of the danger signs during pregnancy and encouraged to contact RHC midwives if they noticed any such signs. We found a clear pattern between family support in receiving antenatal care and the age of the mother-in-law: those aged around 50 and younger encouraged their daughters’-in-law to make regular antenatal visits; mothers-in-law of the older generation saw antenatal care as unnecessary and, in some cases, as harmful to the foetus.

*“My mother-in-law never let me see a doctor. She used to say, all women of her generation delivered at home without any doctors. So I had to go to my gynaecologist secretly: I worked in a farm back then, so I would ask my colleagues to cover up for me at work, would take some money from my husband and go to see a doctor [ … ]”* *–* A 43-year old housewife, 23 years in marriage.

All interviewed women delivered in the maternity hospital located in Isfara town; reported complications during pregnancy or delivery were managed by specialized secondary or tertiary facilities. The costs associated with delivery, the management of complications and hospital stay were in all cases covered by the husband’s family. Crucial decisions such as when to seek obstetric services and whether and how long to stay in a facility were taken by the mother-in-law.

“*All the costs related to my childbirth were covered by my husband’s family. But my mother was so happy to have a grandchild from me that she wanted to share the costs*” – A 21-year old housewife, 3 years in marriage.

### Study group 2: Healthcare providers

We conducted 16 semi-structured interviews with healthcare professionals providing maternity services at primary, secondary and tertiary levels. The characteristics of this study group are presented in Table 2.

**Table 2:** Characteristics of study group 2: Healthcare professionals

		N	%	Min	Median	max
Sex	Female	15	93.8			
	Male	1	6.2			
Age^a^		15	93.8	25	43	62
Position	midwife	8	50.0			
	obstetrician/gynaecologist	5	31.3			
	family doctor	1	6.2			
	director regional RHC^b^	1	6.2			
	head village health facility	1	6.2			
Specialisation/area	midwifery	8	50.0			
	obstetrics/gynaecology	4	25.0			
	family medicine	2	12.5			
	emergency obstetric care	1	6.2			
	antenatal care/genecology	1	6.2			
Level of healthcare facility	regional RHC^b^	4	25.0			
	village RH centre	7	43.8			
	village health centre	2	12.5			
	EmOC^c^ facility	3	18.7			
Years of practice^a^		15	93.8	1	20	35

^a^Data incomplete

^b^Reproductive health centre

^c^Emergency obstetric care

#### Complications in pregnancy and childbirth

Depending on the facility level they represented, respondents dealt with different proportions of antenatal and obstetric complications out of the total number of pregnant women served: from 10% in the primary healthcare facilities to 90% in specialized tertiary facilities. The leading medical causes of complications reported by respondents were anaemia, extra-genital diseases, blood pressure disorders and kidney diseases. A growing rate of genital and venereal infections was associated with the long-term labour migration of the local male population, who allegedly used commercial sex services while abroad.

“*I’m seeing less complications nowadays [ … ] because women started using contraception [ … ] In older times we would go door to door explaining the advantages of contraception, while nowadays women use contraceptives on their own [ … ] Trust in contraception is growing … ”* – Rural midwife with extensive work experience.

All interviewed healthcare providers pointed out a number of non-medical factors that eventually resulted in antenatal or obstetric complications. *Economic*: on the one hand, limited resources led to chronic malnutrition that often resulted in anaemia and compromised the immune status of pregnant women; on the other hand, the burden of healthcare costs inhibited women from obtaining necessary services that could have prevented adverse outcomes. *Cultural:* local practices such as marriage between cousins often resulted in congenital malformation of a foetus and abortion; or the management of pregnancy complications by traditional healers sometimes caused severe haemorrhage. *Social*: traditional gender roles and practices in northern Tajikistan see an individual’s health as a family issue to be managed by the household lead, mainly a mother-in-law. The latter, according to the healthcare providers, often delayed a daughter-in-law’s access to maternity services, which posed a high risk of pregnancy complication and adverse outcomes.

Widely spread myths among elderly women that “gynaecological examination at an early stage of pregnancy causes miscarriage”, or “ultrasound screening of a pregnant woman results in infant’s hypertension” may have sometimes led to adverse pregnancy outcomes that could have been prevented by regular antenatal care. A general strong disbelief in necessity and effectiveness of antenatal care among elderly women who are often the ultimate decision-makers was reported as a significant barrier to access and utilization of maternity services.

#### Caesarean section

Reportedly, the decision to have a caesarean section is among the most difficult that families have to take. Generally seen as a major surgery with multiple adverse effects on a woman’s health and reproductive capacities, the caesarean section is widely disliked and avoided by the local population. When recommended as a risk-mitigation or life-saving measure, a decision to have the caesarean section is never taken by a woman herself; in a family council the final word usually rests with in-laws. Expenses associated with the surgery - planned or emergency - are in most cases covered by a husband’s family, with some instances when a woman’s family shares the costs.

*“In my practice, I’ve never seen a woman who would independently take a decision to have a C-section after I recommend her to do so. First, she goes home to discuss this with her husband, then with a mother-in-law. And since I know that often even husbands have little influence on the final decision, I always ask a woman to invite her mother-in-law to see me … ”–* Gynaecologist, Regional Reproductive Health Centre, Isfara.

#### The three delays

In the course of the interviews, we also asked the healthcare professionals to apply the “Three delays” model to their experience and describe the reasons for antenatal and obstetric complications that result in the delayed access to healthcare services. *Decision to seek care* was unanimously stated as the leading cause for delayed access. With primary healthcare facilities and RHCs functioning in each village, and given the insignificant distance to the hospital in town, *reaching a healthcare facility* was not seen as an obstacle to timely access. *Receiving care* was reported to cause delayed access when patients with complications were referred to the only tertiary-level, overburdened facility in Isfara town.

*“Usually our women with complications are from poor and disadvantaged families [ … ] When emergency obstetric care is needed, we accompany them to the central hospital in town, and that is where often the complication gets worse as the hospital is always flooded with patients [ … ] ”* – Rural midwife, extensive record of practice.

#### Antenatal care reform

Finally, while conducting this study, we have come to know that the recent reform of the antenatal care system and the introduction of family medicine as the primary entry point to services caused certain changes in the antenatal care provision in Tajikistan. Currently, physiological pregnancies without complication are managed by family doctors and midwives, while gynaecologists are involved only for complicated cases. The original aim of this reform was to reduce the burden on the country’s maternity system, which suffers from a shortage of human resources and excess workload. Actual developments in northern Tajikistan, however, demonstrated adverse effects of this reform on the utilization of antenatal services. Traditionally, all maternity specialists in the region have been female, and local women are reluctant to be examined by male health professionals, who constitute almost half of the family doctors. As a result, (a) women choose to skip antenatal care; (b) gynaecologists, who are no longer responsible for physiological pregnancies, have to admit pregnant women upon the request of their male colleagues from the family medicine system.

### Study group 3: Mothers-in-law

Two focus group discussions gathered 16 mothers-in-law aged between 47 and 61.

#### Health beliefs and behaviour

The respondents reported combining conventional medicine with traditional healing rituals; when no immediate effect of medical intervention is seen, religious rituals are additionally performed. Self-medication with pharmaceutical drugs was found to be widespread for conditions such as cough, sore throat, tooth ache, diarrhoea or fever. All women positioned themselves as heads of their families responsible for the health and well-being of their household members, hence the preventive and treatment measures described above equally applied to all family members. None of the respondents reported having healthcare funds separate from those of the family: the necessary amount was taken from the household budget, which was usually formed from the contributions of working men. In case of a pregnancy in the family, the mother-in-law set aside some money for delivery-related costs and to buy necessary supplies for a new-born ahead of time.

*“When there is a health issue in the family, we see a doctor in the first place [ … ] always [ … ] only then can we do the other things[ … ] like seeing a mullah [religious leader]”* – Rural elderly woman, Kulkand.

#### Decision to seek care

Participants unanimously stated that a decision to seek healthcare services for any household member was a family decision primarily discussed with household leads. The mother-in-law considers herself responsible for taking the family member in need to a healthcare facility. Most women expressed their discontent with a growing trend among young women of discussing their health issues with their husbands before consulting with them [mothers-in-law].

*“If I see that her [daughter’s-in-law] children are unwell, I take them to a doctor. You know, we mothers-in-law play a role in the family. We are responsible.”* - Rural elderly woman, Kulkand.

#### Pregnancy in the family

We found that a mother-in-law’s attitude to antenatal care is closely associated with her age: while women aged around 60 see antenatal care as an unnecessary waste of time and resources - and sometimes a harmful practice, - younger women supported their daughter-in-law’s antenatal visits. All mothers-in-law stated a preference for assisted delivery in maternity hospitals; however, most of the women performed religious pre-birth rituals. In general, all women that participated in the discussions considered themselves responsible for both the unborn child and the pregnant woman’s health and well-being.

*“Everyone has hypertension nowadays, even little children* [ … ]. *It’s all because of ultrasound examination during pregnancy* [ ] ***”*** **-** Rural elderly woman, Kulkand.

## Discussion

With this qualitative study we aimed to explore factors that influence rural women in northern Tajikistan to access and use healthcare services. Special emphasis on access *to* and utilization *of* maternity care was instrumental in investigating whether decision-making processes in families may cause antenatal and obstetric complications due to delayed access. The adaptation of the “Three delays” model [20] as the conceptual framework not only served the objectives of our study, but also enabled its potential replication and allowed for comparison of our findings with results from similar studies.

Our study demonstrated that rural women in northern Tajikistan have no or extremely limited autonomy to make independent decisions about their health and well-being. Traditional patterns of family organization and culturally assigned gender roles within family structures oblige married women to reside with their in-laws and/or sometimes other extended family members of their husbands. Decisions to seek and utilize healthcare services by married women are strongly influenced by their mothers-in-law, who were found to act as household leads and ultimate decision-makers when matters of their sons’ families were involved. The revealed pattern of the decision-making process involved several steps and actors: (a) the need to seek health care should be first recognized by household leads; (b) permission to seek and utilize healthcare services should be obtained from at least two family members. In most cases, these were found to be a mother-in-law and a husband, and this hierarchy pertains regardless of whether the husbands are in country or in the long-time labour migration abroad.

These findings resonate with the vast body of evidence from different settings with similar gendered family roles [18, 25–28]*.* Dube [25] in her book analysing gender performance within family structures in Muslim, Buddhist and Christian populations of Bangladesh, Pakistan, India, Nepal, Malaysia, Sumatra, Thailand and Philippines, concludes that a woman in those cultures is seen in the shadow of an imaginary mother-in-law. Other studies on the influence of mothers-in-law on family planning decisions in rural India [28] and Pakistan [29] highlight the dominating role of mothers-in-law in the fertility issues of their children’s families.

We found that mothers-in-law base their decisions primarily on their own experiences of illness and utilization of care, especially where pregnancy and childbirth are concerned. If a healthcare service or intervention is not perceived as necessary or adequate by mothers-in-law, they tend to use their power to prevent their family members from using it. It particularly applies to antenatal care, which is widely seen as unimportant and, in some instances, as harmful to a foetus. This finding is supported by the results of the qualitative research on the role of mothers-in-law in the uptake of antenatal care in Nepal [30] and the quantitative study on the relationship between women’s autonomy and maternal care utilization in India [26], showing that decisions on the use of antenatal care influenced by mothers-in-law do not always reflect the actual needs of pregnant women.

In addition, a number of myths and misconceptions about routine pregnancy-related interventions, such as ultrasound and gynaecological examination common among the elderly generation in Isfara district, were found to prevent pregnant women from using antenatal services. This is consistent with the existing evidence on how knowledge and beliefs around pregnancy and childbirth influence actual health seeking practices. Goodburn and colleagues [31], in their research on the effect of beliefs and practices on maternal morbidity and mortality in Bangladesh, found the neglect of health services as a factor associated with maternal morbidity and mortality. Khadduri and colleagues [32] revealed that, even knowing the benefits of maternity care, women in Haripur (Pakistan) opt for traditional practices harmful for women and new-borns.

Our results show that *reaching a healthcare facility* and *receiving necessary care* do not pose notable obstacles to obtaining health services. Primary healthcare facilities, including antenatal and obstetric care, exist in all villages; secondary and tertiary care can be obtained in Isfara town, which is located 15 to 50 min away by car. While all respondents reported being satisfied with the primary health care services they received in their villages, referral to secondary, tertiary or specialized services (laboratory tests, advanced diagnostics) in Isfara town was associated with reportedly insignificant issues of traveling time and cost of services.

Whether a husband was in the country or absent did not influence a woman’s autonomy or position to take decisions on seeking health; the primary role of a mother-in-law as a household lead and decision-maker was preserved regardless of a husband’s whereabouts. However, in the periods when their husbands were in the country, women reported having better access to financial resources and support.

The study has some important policy implications. First, given a relatively high rate of pregnancy and childbirth related complications due to delayed access to care in a setting with well-established antenatal and obstetric care structures, clinical standards and medical personnel, non-structural determinants of ill-health such as cultural beliefs and practices should be recognized by the decision-makers in the health sector. In doing so, it will be important to (a) support research on non-structural determinants of women’s access to and utilization of health care; (b) develop response measures, including effective awareness raising and behaviour change communication strategies on the community level; (c) introduce necessary changes to maternal health policies in order to ensure effective and sustainable implementation of the selected response measures.

Second, deteriorating socio-economic conditions and education opportunities of girls and women over the last two decades [7]*,* coupled with growing radical Islamic tendencies in Tajikistan may have a drastic effect on women’s position in society. Comprehensive, intersectoral actions to empower women and increase their autonomy and possibilities to attain the highest level of well-being and health should be urgently prioritized.

The antenatal care reform and introduction of the family medicine as the primary entry point to maternity services were seen by the interviewed healthcare professionals as a negative development. In addition to the increased risks of antenatal and obstetric complications, the reform is believed to exacerbate the inefficiency of the antenatal care system in the region. While family doctors paid by the system for provision of antenatal services fail to cover pregnant women with antenatal care, gynaecologists who are no longer responsible for physiological pregnancies often have additional workload due to the reluctance of pregnant women to be seen by a male family doctor. In addition, maternity hospitals report that women admitted for childbirth (whose antenatal care was covered by family doctors) often have incomplete or inadequately managed antenatal records, with crucial pregnancy-related data missing.

Building on the findings of this study that women’s access to health care services in Tajikistan is strongly associated with a number of non-structural determinants, more research looking at factors influencing the access and utilization of care of both, other groups of women and other regions of the country would provide a basis for large-scale interventions to improve the health and well-being of women in Tajikistan.

Our study has certain limitations. This was a small-scale study in one district of Sughd Region, hence not all results can be generalized to similar groups of women in other regions of Tajikistan. Data were collected in Tajik or Russian and analysed in English, which may have led to a certain distortion of content as a result of translation. Finally, keeping in mind the cultural inappropriateness of publicly disclosing family or personal issues, which could possibly have led respondents to provide socially acceptable answers rather than sharing actual experiences, we applied a certain level of reservation when analysing and triangulating our data.

## Conclusions

This study suggests that a traditional pattern of making decisions on seeking antenatal and obstetric care in northern Tajikistan may often cause delays in accessing necessary maternity services and result in adverse health outcomes for women. It also demonstrates that women in rural Tajikistan have limited control over their health and well-being in the reproductively active period of their lives. Decisions about their health and family planning choices may often be based not on the best available and appropriate options but rather culturally expected grounds. Women may often find themselves in a situation where they are unable to take the independent decision about their own body. Recognition of these findings by respective decision makers, development and implementation of effective response measure, and revision of maternal health policies to incorporate cultural determinants of access to care can contribute to reducing the numbers of preventable complications in pregnancy and childbirth in Tajikistan as well as in countries with a similar cultural background.

On the service provision side, a practical solution for male family doctors providing antenatal care should be found to ensure that women’s reluctance to receive antenatal services from male family doctors does not prevent them from accessing necessary care during pregnancy.

## Abbreviations

EmOC: Emergency obstetric care
IDI: In-depth interview
FGD: Focus group discussion
RHC: Reproductive Health Centre
SSI: Semi-structured interview
